# Data projections of the replacement ratios for the cohorts of retirees in Chile under different policy reforms

**DOI:** 10.1016/j.dib.2022.108516

**Published:** 2022-08-05

**Authors:** Carlos Madeira

**Affiliations:** Central Bank of Chile, Chile

**Keywords:** Pensions, Social security system, Income redistribution, Contributory pensions, Retirement age

## Abstract

This article provides five panel datasets for the projections of the mean replacement ratios of pension income relative to the worker's income. The time dimension is from year 2022 until 2055. The panel groups consider the gender, income and education of the workers. Furthermore, the variables consider different scenarios for the social security system: (1) a baseline with the current policies, (2) an increase of retirement age to 67 years, (3) an increase of the retirement age to 67 years and a 6% increase of the contribution rate, with a rate of 0–3% going to solidarity funds.

## Specification Table

**Table:** 

Subject	Economics, Econometrics and Finance.
Specific subject area	Household Finance. Pensions. Social security. Labor Economics.
Type of data	Table (Excel format and Stata dta files)
How data were acquired	Data uses publicly available raw data from the Chilean Family Expenditures Survey (EPF, 2017 wave) to calibrate the future labor income and pension contributions of each worker until retirement.
	Hardware: data analysis was performed in a standard notebook with an Intel Core i7-4700HQ 2.40 GHz processor with 16.0 GB of RAM. Software: Stata MP-6 (version 15.1).
Data format	Analyzed
Description of data collection	Data consists of different panel groups (given by the gender, education, and income of the workers) and the replacement ratios of retiree income cohorts at a yearly frequency between 2022 and 2055. The datasets consider 6 different scenarios for the future public policy. The time horizons in which the policies are analyzed start in 2022 (because the last Chilean pension policy was implemented in January of 2022) and end in 2055 (which is when the youngest workers of the present period start retiring and the alternative pension policies will have a full effect).
Data source location	Institution: Instituto Nacional de Estadísticas (INE, in English, Bureau of Official Statistics) City/Town/Region: Santiago Country: Chile https://www.ine.cl/estadisticas/sociales/ingresos-y-gastos/encuesta-de-presupuestos-familiares (EPF)
Data accessibility	With the article. Repository name: Mendeley Data Data identification number (DOI): 10.17632/59j9wkwvd8.1. Direct URL to data: https://data.mendeley.com/datasets/59j9wkwvd8/1
Related research article	C. Madeira, The impact of the Chilean pension withdrawals during the Covid pandemic on the future savings rate, J Int Money Finance, 126 (2022) 102,650.

**Value of the Data**

• The data can be used to study the forecasted Chilean retirees’ pension income as a replacement ratio of their last labor earnings over the period between 2022 and 2055 (Madeira [1]). Such studies can be done by demographic groups (by gender, income or education) and over time at an annual frequency until 2055.
• The data shows how the replacement ratios are affected by different policies such as an increase in the contribution rate and delaying of the retirement age.
• Since the pension system represents a large component of national wealth and savings in most countries (OECD [5]), the data can contribute to the study of the low savings rate in Latin America (Cavallo and Serebrisky [2]) and the policies necessary to solve this problem in Chile.

## 1 Data

The dataset provides projections of the replacement ratios of retirement income relative to the last income of the worker (Table 1). Average values are reported for the cohorts of future retirees in Chile from 2022 until 2055. The time of the analysis starts in 2022, because the last Chilean pension policy was substantially changed due to recent policies, such as the three pension withdrawals (August of 2020, December of 2020, April of 2021) and a non-contributory public pension increase in January of 2022 (Madeira [1]). The data ends in 2055 when the different policy alternatives reach a full effect.

**Table 1:** Description of the dataset (SRates_Dib.xlsx, plus its Stata .dta version) provided in this article.

Analyzed dataset	Description
ReplacementRatios_X1.xlsx (ReplacementRatios_X1.dta)	Forecasts for the period 2022–2055 of the replacement ratios of each new cohort of retirees. The cohorts consider the entire population of new retirees.

ReplacementRatios_X2.xlsx (ReplacementRatios_X2.dta)	Forecasts for the period 2022–2055 of the replacement ratios of each new cohort of retirees, with cohorts by gender.

ReplacementRatios_X3.xlsx (ReplacementRatios_X3.dta)	Forecasts for the period 2022–2055 of the replacement ratios of each new cohort of retirees, with cohorts by gender and education (5 categories of worker completed education).

ReplacementRatios_X4.xlsx (ReplacementRatios_X4.dta)	Forecasts for the period 2022–2055 of the replacement ratios of each new cohort of retirees, with cohorts by gender and national labor income quartile (4 categories).

ReplacementRatios_X5.xlsx (ReplacementRatios_X5.dta)	Forecasts for the period 2022–2055 of the replacement ratios of each new cohort of retirees, with cohorts by gender, education (5 categories) and national labor income quartile (4 categories).

Projections are given for 6 different scenarios: “Current policies,” “Increase of retirement age to 67 years,” “Increase in age plus 6% increase in personal contribution rate,” “Increase in age, 5% increase in contribution rate, 1% solidarity tax rate,” “Increase in age, 4% increase in contribution rate, 2% solidarity tax rate,” “Increase in age, 3% increase in contribution rate, 3% solidarity tax rate”. There are 5 datasets which express average panel time series for 5 different populations: (X1) All workers, (X2) by gender, (X3) by gender and worker income quartile, (X4) by gender and education, (X5) by gender, education, and income quartile. The same datasets are available in both Excel and Stata formats.

This is the list of variables available in the panel datasets, with a summary description. The list is organized according to 3 categories of variables: (1) panel groups (demographic groups by gender, education, income or the whole population of new retirees), (2) time variable (year of retirement of the cohort of new retirees), and (3) the replacement ratios of retirement income relative to the last wage, according to the different public policies adopted for the pension system.

Demographic variables (gender, education, national income quartile):

These variables describe the cohort of retirees in terms of their gender and education.

(1) Pop “Entire population” (this variable always takes a value of 1).
(2) Sexo “Gender of the worker (1 Male, 2 Female)”.
(3) Educ_ecf “Education level of the respondent,” with values 1 “Elementary education” 2 “Secondary education” 3 “Technical or Some college” 4 “College education” 5 “Post-graduate education”.
(4) Quartile “Worker income quartile: 1 lowest income workers (25% lowest earnings workers), 2 (workers between the 25 and 50% of highest labor earnings), 3 (workers between the 50 and 75% of highest labor earnings), 4 highest income (workers with the 25% highest labor earnings).”

Year of the retirement for each cohort:

This variable corresponds to the year in which each cohort of workers enters retirement, that is, the first year in which the retirees receive their pension.

(1) YearR “Year of retirement for each cohort (yearly frequency between 2022 and 2055)”.

Replacement ratios of retirement income relative to worker's last income according to the public policy scenario adopted between 2022 and 2055:

These variables correspond to the average replacement ratio of the pension income of each cohort of retirees during their first year of retirement relative to their labor income during their last working year. The alternative policies analyzed include the current policies as a baseline, an increase of the retirement age to 67 years, plus policies that increase the personal contribution (between 3 and 6%) and the solidarity tax rate (between 0 and 3%) in addition to the increase of the retirement age.

(1) rr_ratioTot_B “Current policies”.
(2) rr_ratioTot_S1 “Increase of retirement age to 67 years”.
(3) rr_ratioTot_S2 “Increase of retirement age to 67 years plus 6% increase in personal contribution rate”.
(4) rr_ratioTot_S3R2 “Increase of retirement age to 67 years, 5% increase in contribution rate, 1% solidarity tax rate”.
(5) rr_ratioTot_S4R2 “Increase of retirement age to 67 years, 4% increase in contribution rate, 2% solidarity tax rate”.
(6) rr_ratioTot_S5R2 “Increase of retirement age to 67 years, 3% increase in contribution rate, 3% solidarity tax rate”.

## 2 Experimental Design, Materials and Methods

The data consists of a simulation of the future contributory pensions plus public solidarity benefits for a sample of Chilean households (Madeira [1]), with mean forecasts presented for each new cohort of retirees between 2022 and 2055. The panel groups are given by gender, education and income.

The data uses the pooled cross-section sample of households from the Chilean Family Expenditures Survey (in Spanish, *Encuesta de Presupuestos Familiares*, hence on EPF) wave of 2017. The calibration estimates the worker's average yearly labor income using their job income while working and their average time in unemployment, with unemployment risk given according to 538 workers’ types which are obtained from the multivariate vector of the workers’ sex, age, education, industry and region (Madeira [3]). The life expectancy and population weights of workers of each gender (male, female) with different ages are then adjusted over time using population forecasts from the United Nations (Madeira [1]).

The methodology starts by first computing the permanent income of each worker *k* at time t ($P_{k,t}$), according to its average time employed ($1-u_{k,t}$) versus unemployed ($u_{k,t}$):

(1) $$P_{k,t}=G_{k}W_{k,t-1}\left(1-u_{k,t}-u_{k,t}RR_{k}\right),$$

with $G_{k},W_{k,t},u_{k,t},RR_{k}$ denoting, respectively, the yearly growth rate of labor income in the occupation of worker *k*, the wage of worker *k* in its occupation in the previous year, the unemployment probability of *k*’s worker type, and the replacement ratio of labor income during unemployment. These components of the model are obtained for 538 workers’ types which are obtained from the multivariate vector of the workers’ sex, age, education, industry and region (Madeira [3]).

I then obtain the worker's contributions ($PWI_{k,t}$) to the social security retirement system until age S(t,k):

(2) $$PWI_{k,t}=\sum_{h=25}^{S\left(t,k\right)-1}cr_{h}r_{h}\min\left(mc_{h},P_{k,h}\right)pc_{k,h}-r_{2021}pw_{k}^{1+2+3}$$

with $cr_{h},r_{h},mc_{h},P_{k,h},pc_{k,h}$ denoting, respectively, the contribution rate, the average rate of return between time h and time t, the top contributory income value, the permanent income, and the probability of contribution (given by the probability of being both in the labor force and in forma work) at time h. $pw_{k}^{1+2+3}$ denotes the wealth withdrawn by each worker from its pension account during the pandemic.

When worker *k* at age $R_{k}$ in year t, its accumulated pension turns into a monthly annuity for their life, $pa_{k,t}=\frac{rPWI_{k,t}{\left(1/\beta \right)}^{R_{k}-S\left(t,k\right)}}{1-{\left(1/\beta \right)}^{-12\left(T_{k,t}-R_{k}\right)-S\left(t,k\right)}}$, with $T_{k,t},\beta ,r=\left(\frac{1}{\beta }\right)-1$ denoting, respectively the worker's life expectancy, the discount factor and the risk neutral interest rate.

Each member of a family within the nine lowest deciles of income ($SB_{k}$) is also entitled to solidarity benefits according to the law of 2022 ($B_{k,t}$) and to additional solidarity benefits funded from the “solidarity tax rate” that may be implemented in the future.

The new solidarity benefits of each retiree k can therefore be expressed as

(3) $$B_{k,t}=b_{1}1\left(pa_{k,t}<b_{2}\right)+b_{1}\left(1-\frac{pa_{k,t}-b_{2}}{b_{3}-b_{2}}\right)1\left(b_{2}<pa_{k,t}<b_{3}\right),$$

with $b_{1}=185,000$, $b_{2}=630,000$, and $b_{3}=1,000,000$. Therefore, retirees receive 185,000 pesos as solidarity benefits if their contributory pension income is below 630,000 pesos and then receive gradually fewer solidarity benefits until their contributory pension income is already one million Chilean pesos.

The total pension $TP_{k,t}$ is therefore given by:

(4) $$TP_{k,t}=pa_{k,t}+SB_{k}\left(B_{k,t}+SP_{k,t}\right),$$

with $SP_{k,t}=\frac{rSPT_{k,t}{\left(1/\beta \right)}^{R_{k}-S\left(t,k\right)}}{1-{\left(1/\beta \right)}^{-12\left(T_{k,t}-R_{k}\right)-S\left(t,k\right)}}$ and $SPT_{k,t}=\sum_{h=0}^{T_{k,t}-R_{k}}\beta^{h}\left(MP_{t}-\left(pr_{k}-1\right)\right)$ denoting, respectively, the new solidarity benefits received by worker k retiring in year t and the total discounted value of all the new solidarity benefits that this worker will receive during its life in retirement. In this formula, $MP_{t}=\frac{SC_{t}}{NR_{t}}+\frac{mp-1}{2}$, with $SC_{t},\;NR_{t}$ denoting, respectively, the total funds collected through the new solidarity tax in each year and the total number of retirees receiving solidarity benefits. In this expression mp = 10,001 is the total number of groups among whom the solidarity funds are distributed and $pr_{k}$ is the ranking of the group of retiree *k* among the total number of retirees that can benefit from the solidarity system. This mechanism ensures that more solidarity funds are distributed towards those of lowest income.

The calibration of the permanent income requires estimating the yearly growth rate of labor income in the occupation of worker *k*, the wage of worker *k* in its occupation in the previous year, the unemployment probability of *k*’s worker type, and the replacement ratio of labor income during unemployment. These heterogeneous parameters were obtained from the rotating panel samples of the Chilean Employment Survey (Madeira [3]) from the labor earnings of each individual worker i of the sample in year t ($y_{i,t}$), the characteristics of each worker ($x_{i}=x_{k}$) and their unemployment status ($U_{i,t}=1$):

(5) $$G_{k}=\frac{E\left[y_{i,t}|t=h,x_{i}=x_{k}\right]}{E\left[y_{i,t}|t=h-1,x_{i}=x_{k}\right]}-1;$$

(6) $$W_{k,t}=\max\left(y_{i,y},E\left[y_{i,t}|t=h,x_{i}=x_{k}\right]\right);$$

(7) $$u_{k,t}=\text{Pr}\left(U_{i,t}=1|t,x_{i}=x_{k}\right);$$

(8) $$RR_{k}=\frac{E\left[y_{i,t}|t=h,U_{i,t}=1,x_{i}=x_{k}\right]}{E\left[y_{i,t}|t=h,U_{i,t}=0,x_{i}=x_{k}\right]}.$$

These parameters are obtained for each year of the Chilean Employment Sample since 1990 until now for the workers’ types given by the multivariate vector x_k_ of the workers’ sex, age, education, industry and region (Madeira [3]). Finally, the probability of contribution (given by the probability of being both in the labor force and in forma work) at time t is also obtained from the Chilean Employment Survey. The calibration of the worker's career paths for the future years is kept similar to the year 2017 (which had the lowest unemployment rate and is therefore close to full employment), but the parameters take into account the aging of the workers.

Finally, to calculate the savings and pensions for each cohort retiring between 2018 and 2055, each year uses the life expectancy estimates after age 60 for each gender in Chile to obtain $T_{k,t}$ and adjusts the population weights as follows: $w_{k,t}=w_{k}^{EPF}Pop_{t}\left(s_{k},age_{k}\right)/Pop_{2017}\left(s_{k},age_{k}\right)$, with $w_{k}^{EPF}$denoting the original EPF weights in 2017 and $Pop_{t}\left(s_{k},age_{k}\right)$ being the number of people in each sex-age bracket. Life expectancy $T_{k,t}$ and population by sex-age $Pop_{t}\left(s_{k},age_{k}\right)$ for each year t are obtained from United Nations estimates.

Users can download (at no cost) the raw data of all the EPF surveys, including the 2017 wave, from the website of the Chilean Institute of National Statistics.

EPF: https://www.ine.cl/estadisticas/sociales/ingresos-y-gastos/encuesta-de-presupuestos-familiares.

The datasets are available at Mendeley Data (Madeira [4]): data.mendeley.com/datasets/59j9wkwvd8/1.

## CRediT authorship contribution statement

**Carlos Madeira:** Conceptualization, Methodology, Software, Validation, Formal analysis, Investigation, Resources, Data curation, Writing – original draft, Writing – review & editing, Visualization, Supervision, Project administration, Funding acquisition.

## Declaration of Competing Interest

The author declares that he has no known competing financial interests or personal relationships which have or could be perceived to have influenced the work reported in this article. I received no funding from any institution besides my employer which is the Central Bank of Chile. Furthermore, there are no patents or impediments to publication, including the timing of publication, with respect to the intellectual property of the article or the associated dataset.

## Data Availability

Projections of the replacement ratios for the cohorts of retirees in Chile for the period 2022 until 2055 (Original data) (Mendeley Data).
